# Modelling the Effect of Compliance with Nordic Nutrition Recommendations on Cardiovascular Disease and Cancer Mortality in the Nordic Countries

**DOI:** 10.3390/nu11061434

**Published:** 2019-06-25

**Authors:** Sanjib Saha, Jonas Nordström, Irene Mattisson, Peter M. Nilsson, Ulf-G Gerdtham

**Affiliations:** 1 Health Economics Unit, Department of Clinical Science (Malmö), Lund University, SE-22381 Lund, Sweden; ulf.gerdtham@med.lu.se; 2 School of Economics and Management, Agrifood Economics Centre, Lund University, SE-22007 Lund, Sweden; jonas.nordstrom@agrifood.lu.se; 3 Department of Food and Resource Economics, University of Copenhagen, DK-1958 Frederiksberg C, Denmark; 4 National Food Agency, SE-75126 Uppsala, Sweden; evairene@live.se; 5 Department of Internal Medicine, Skane University Hospital, SE-20502 Malmo, Sweden; peter.nilsson@med.lu.se; 6 Department of Clinical Sciences (Malmo), Lund University, SE-20502 Malmo, Sweden; 7 Department of Economics, Lund University, SE-22363 Lund, Sweden

**Keywords:** Nordic diet, Nordic countries, dietary guidelines, macro simulation model, cardiovascular diseases, recommended intake, health Benefit

## Abstract

The objective of this study is to estimate the number of deaths attributable to cardiovascular diseases and diet-related cancers that could be prevented or delayed in the Nordic countries, i.e., Sweden, Denmark, Finland, Norway, and Iceland, if adults adhere to the Nordic Nutrition Recommendations (NNR). A sex- and age-group specific epidemiological macro-simulation model was used to estimate the preventable deaths due to the differences between country specific actual intake and recommended intake of changes in food components. Data included in the model are a baseline scenario (actual dietary intake), a counterfactual scenario (recommended intake), and age-and sex-specific mortality for cardiovascular and diet-related cancer diseases, together with the total population risk of a specific year. Monte Carlo analyses with 5000 iterations were performed to produce the 95% uncertainty intervals. The model predicts that Iceland would benefit the most by adhering to the NNR, followed by Finland. In all the Nordic countries, the highest benefit would be achieved by adhering to the fruits and vegetable intakes, except Denmark, where a lower recommended intake of salt would provide the highest benefit. For men, fruits and vegetables could have saved more lives compared to other dietary components for all the Nordic countries, while for women, dietary fiber was the most prominent factor, except in Iceland. The Nordic Council should consider policies for promoting healthy eating according to the needs of each country.

## 1. Introduction

For several decades, the Nordic countries have collaborated to establish dietary guidelines. The Nordic Nutrition Recommendations (NNRs) are based on research data from epidemiological studies and laboratory studies by a panel of experts. These recommendations are applicable to all the Nordic countries: Sweden, Denmark, Finland, Norway, and Iceland [1]. The decision to develop joint NNRs by the Nordic countries emerged not only from the geographical location of the Nordic countries but also from the similarities shared in dietary habits and similarities in the prevalence of diet-related diseases, such as cardiovascular diseases (CVDs), obesity, and Type 2 Diabetes (T2D) [2]. The NNR include reference values for total energy intake and recommendations on macronutrients as a percentage of total energy intake, daily intakes of vitamins and minerals, as well as intake of fiber and salt, together with recommendations on physical activity [1].

The latest NNR was developed in 2012, when about 100 scientists from all five Nordic countries were involved in developing recommendations [1]. The primary aim of the NNR 2012 was to present the scientific background of the recommendations and their applications. A secondary aim was to function as a basis for national recommendations, i.e., food-based dietary guidelines that are adopted by the individual Nordic countries. Following these, the Nordic countries have developed their own dietary recommendations. However, studies exploring how well the Nordic population adheres to dietary recommendations are limited. For example, there is a large gap between the actual dietary practice and recommended intake in Sweden [3]. The SYSDIET study found that 65% of the study participants who were from Sweden, Finland, Denmark, and Iceland did not meet the recommendations for saturated fatty acids, polyunsaturated fatty acids (PUFA), dietary fiber, and sodium [4]. In Norway, less than 40% of the adolescents adhere to recommendations for frequencies of eating fruits, vegetables, added sugar, and fish [5]. Adherence to dietary recommendation is also low for the general Danish population [6].

The number of deaths from chronic diseases and/or the incidences of chronic diseases that could be prevented or avoided by changing the dietary intake of the Nordic population according to the recommendations of the NNR 2012 are still unidentified. Furthermore, the dietary components that could provide the highest beneficial health effects in the respective countries are unknown. Since most of the top determinants of the burden of disease are diet-related such, as CVDs, T2D, obesity, and numerous cancers [7], it is thus important to identify and measure the health benefits (losses) that can be obtained (avoided) if the Nordic populations adhere to the NNR 2012. Such knowledge could guide policymakers to prioritize interventions to allocate scarce resources strategically, since these diseases contribute to a substantial economic burden and create health inequalities [8,9] in these societies.

Simulation models are suitable for integrating results from observational studies where different experimental studies, such as randomized controlled trials (RCTs), are difficult to conduct [10,11]. This is more suitable for diet-related intervention/policies, where it is impractical or unethical to estimate, for example, the effect of the proposed taxes on saturated fat intake by RCTs followed over, say, 50 years. This would mean randomizing people, shops, or areas to face increases in food prices. A simulation model is a helpful tool that can combine all the available evidence to estimate a scenario where a head-to-head comparison is impossible to perform. Simulation models use a collection of mathematical equations to quantify the relationships between proposed or hypothetical interventions and specific outcomes of interests [12]. Moreover, simulation models pose many advantages over RCTs, for example, linking intermediate clinical endpoint to final outcomes, e.g., linking changes in blood pressure to hypertension-associated diseases. This enables policymakers to make decisions in the absence of reliable data using realistic assumptions [13].

The aim of this study is firstly to quantify health benefits by means of deaths related to CVDs and cancer that could be prevented or delayed if the Nordic population could follow the Nordic nutrient recommendations using a simulation model. Secondly, the aim is to perform an inter-country comparison to identify which dietary components would provide the highest health benefits for each country population. Thirdly, this study aims to observe heterogeneity in the quantified benefits based on age and gender.

## 2. Materials and Methods

We compared the recommended nutrient intake with the actual dietary intake of the country-specific Nordic population. A validated and transparent macrosimulation model, the PRIME (Preventable Risk Integrated ModEl) [14], was used to estimate the cardiovascular and diet-related cancer death toll that could be prevented or delayed for the populations of Nordic countries in a year.

### 2.1. Recommended Intake

The NNR 2012 [1] was used as a recommended intake for this study. Each Nordic country has modified the NNR 2012 to fit the food culture and ability of the consumer. The Swedish National Food Agency (Livsmedelsverket) published the revised version of the National Food-based Dietary Guidelines in 2015 (Swedish: Hitta ditt sätt) [15]. The ministry of Food, Agriculture, and Fisheries of Denmark published the official dietary guidelines (Danish: De Officielle Kostråd) for the Danish population in September 2013 [16]. The Norwegian Directorate of Health published a revised version of the Guidelines in 2014, named “Norwegian guidelines on diet, nutrition, and physical activity, 2014” (Norwegian: Anbefalinger om kosthold, ernæring, og fysisk aktivitet, 2014) [17]. The National Nutrition Council led the development of Finish Recommendations together with various stakeholders, Finnish nutrition recommendations 2014 (Finnish: Terveyttä ruoasta. Suomalaiset ravitsemussuositukset 2014) [18]. The official name of the dietary recommendations of Iceland is Dietary guidelines, for adults and children from two years of age (Icelandic: Ráðleggingar um mataræði fyrir fullorðna og börn frá tveggja ára aldri), which was published in 2014 [19]. The guidelines were developed by an expert group, including professionals from academia and the Directorate of Health. For the ease of comparison, we used the same recommendations for each country. The NNR provides the recommendation for energy yielding nutrients as a range. For example, 10%–20% of the energy should come from monounsaturated fatty acids (MUFA). For this study, we required exact targets for consumption, so we converted these ranges into the best values. Furthermore, the NNR guidelines combine fruits and vegetable recommendations into one (i.e., 500 g/day), but we have separated the intake by 250 g for each component in this study.

### 2.2. Actual Intake

The diet in the Nordic countries is characterized by a higher consumption of animal, processed, and sweetened foods, including non-alcoholic beverages and soft drinks [20]. The food consumption patterns in the Nordic countries are also high in dairy and bread [21]. Typically dominating grains are ray, barley wheat, and oats, which are rich in dietary fibers [22]. Saying that, inter-country differences do exist in terms of dietary intake. The actual average dietary intake of Nordic populations was obtained from the most recent dietary surveys conducted in each country. In Table 1, we present the details based on the dietary surveys of Sweden [23], Denmark [24], Finland [25], Norway [26], and Iceland [27]. These surveys were used to estimate population intake of energy, fruits and vegetables, fiber, salt and fats, which includes total fats, saturated fats, PUFA, MUFA, and cholesterol, stratified by age and sex. 

It is not surprising to note that the national dietary surveys vary with respect to sample size, age group, participation rate, as well as the methodology to collect the dietary intakes. Except for Finland, all countries had invited sample representatives to the country. However, the participation rate varied from 36% to 68.8%, where Sweden had the lowest and Iceland had the highest rate of participation. The methods to collect the dietary information also varied. Only Norway and Iceland used a similar method (2 × 24 h recall together with a Food Frequency Questionnaire). The food intake was converted into specific nutrient intakes using country-specific nutrient databases, which are also presented in Table 1.

The actual mean intakes of nutrients is presented in Table 2, together with the recommended intake from the NNR 2012 that was used in this study. However, details of the data used as model inputs are presented in the Supplementary Materials (Annex 1).

### 2.3. The Simulation Model

A comparative risk assessment macrosimulation model, PRIME (Preventable Risk Integrated ModEl) [14] has been used for this study. This model simulates the effect of changes in consumption of foods (fruits and vegetables) and nutrients (dietary fiber, salt and fatty acids) through risk factors, such as serum cholesterol, blood pressure, and overweight/obesity to diet-related mortality from CVDs and diet-related cancers. In a technical report, the details regarding the underlying assumptions of the model are available [14].

To be included in the model, food components have to be recognized as statistically associated with CVD outcomes and cancer, or biological risk factors for these diseases. Meta-analyses obtained from prospective cohort studies and randomized controlled trials are used to parameterize changes in nutritional risk factors and mortality as a result of the change in the population’s intake of food items and nutrients [14]. PRIME estimates the differences in mortality in one single year between the baseline scenario (actual dietary intake, in this case) and the counterfactual scenario (recommended dietary intake).

The model is based on a number of key assumptions:

1. The counterfactuals are based on changing dietary variables that are continuous (e.g., fruit consumption (g/day)), rather than binary exposures (meet recommendations for fruit (yes/no)). Therefore, a distribution of each variable within the population is used as a baseline for the model. For the counterfactuals, a shift of distribution is made so that the new mean level of consumption matches the recommendation, but the variance in the population remains the same as the baseline. This is equivalent to everyone in the population making the same changes to their diet, implying that approximately 50% of the population will still not meet the recommendations in the counterfactual scenario, but this is appropriate since population-level targets (such as dietary recommendations) are monitored by tracking a population’s mean consumption levels.
2. Combined changes in the risks for individuals are multiplicative. For example, if one extra serving of fruits reduces the risk of CVD by 11% and reducing salt intake by 1 g per day reduces the risk by 10%, then both of these behavior changes jointly reduce the risk of CVD death by 19.9% (1 − (1 − 0.11) × (1 − 0.10)). The PRIME model accounts for competing risks by combining relative risks multiplicatively. However, the model is unable to account for interactions between risk factors (e.g., if increasing fruit and vegetable consumption provides more health benefit for low-fiber consumers than high-fiber consumers).
3. Another assumption is that changes in risk follow a log-linear, dose-response relationship, except for obesity, which follows a J-shaped curve. For example, a change in the consumption of fruits and vegetables from 3 to 4 servings has the same effect on relative risk as a change in consumption from 6 to 7 servings. However, an upper threshold was included, above which there are no additional health benefits. The upper thresholds are based on the range of data collected in the meta-analyses used to parameterize the models. It is unlikely that the effects of different food components are independent and additive. By combining parameters multiplicatively, the PRIME model estimates the overlap in estimated changes in the risk of cause-specific mortality as they relate to changes in different dietary components (i.e., the outcome of changing several dietary components simultaneously is less than the sum of its parts and can never exceed 100% risk reduction).

We also assume that Nordic people are similar in all aspects other than food intake, for example in terms of other health-related behaviors, such as physical exercise, alcohol drinking, and smoking habits, although within-country differences do prevail in health behaviors [28]. PRIME has been previously used to answer similar research questions in and France [29], Canada [30], the UK [31], and Sweden [32].

### 2.4. Population Statistics

The model requires age- and sex-specific population mortality for specific diseases for a given year. The mortality data for diet-related cancers (ICD-10: C00-14, C16, C23, and C33-34), coronary heart diseases (ICD-10: I20-25) and stroke (ICD-10: I60-69) were obtained from country specific national databases. This model also required the age- and sex-specific number of populations in that country for that specific year. The population statistics were obtained from the official statistical websites of each country. The latest population and mortality data (from 2016) were used for all the countries except Norway, where the population and death statistics are from 2013.

### 2.5. Uncertainty Analysis

A Monte Carlo simulation was conducted to estimate the Uncertainty Intervals (UI) around the results. Each of the estimates in the model were allowed to vary according to the distribution reported in the accompanying literature. The 95% UI estimates are based on the 2.5th and 97.5th percentiles of results obtained from 5000 iterations of the model.

## 3. Results

The reported intake of fruits and vegetables was lowest for Finish men and women among all Nordic countries (Table 2). Women consumed more fruits than men consumed in all the countries. Women also consumed more vegetables than men consumed, except in Norway and Iceland. Danes had the highest intake of fiber, whereas Icelanders had the lowest intake of fiber. Men consumed more salt than women in all the countries and for both groups, the intake of salt was higher than the recommended intake level (6 g/day). The salt intake was highest among the Danish men and women compared to the other Nordic Countries. Saturated fat intake was higher than the recommendation in all the countries, and Icelanders had the highest intake of saturated fat. The MUFA and PUFA intake was within the range of recommendations but lower than the optimum value (Table 2).

**Figure 1:**
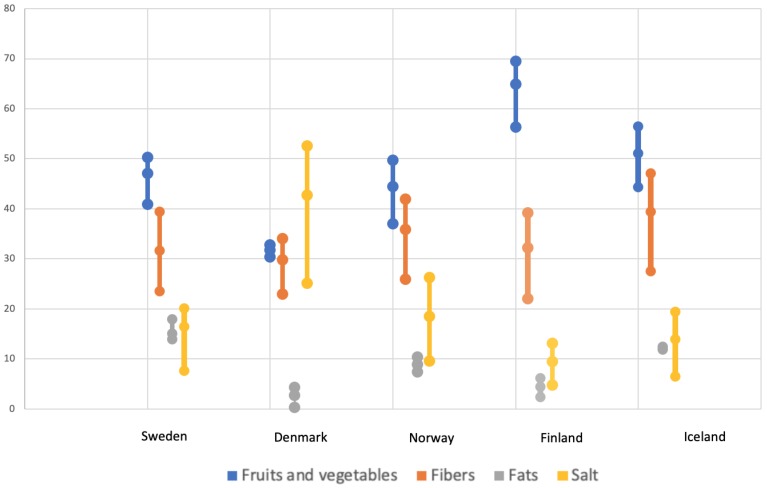
Percentage of deaths (together with a 95% Uncertainty Intervals) delayed or saved by dietary changes in a year in the Nordic Countries.

The model estimates that the highest number of deaths that could be prevented by following the recommendation intake is for Iceland, where 19.7% of the deaths can be prevented, followed by the Finish population (18.9%) (Table 3). In terms of food groups, the highest percentage of deaths can be saved by following the recommendations of fruit and vegetable intake for all the countries except Denmark (Figure 1). For Danes, following salt recommendations could prevent 42.74% of the deaths related to the dietary intake. For the Fins, 64.95% of the deaths could be prevented by following the dietary recommendations for fruit and vegetable intake.

In terms of gender, more deaths could be prevented or delayed among men than women (Table 3). Modifying dietary intake to meet fruit and vegetable recommendations could save more lives for men than for women in all the Nordic countries. This is also true for salt intake; more lives of men than women could be saved by consuming the recommended salt intake. However, women would benefit more from consuming more fiber, except in Iceland.

Most of the deaths that could be prevented or delayed by improving dietary intake are related to coronary heart diseases, followed by stroke in all the Nordic countries (Supplementary Materials, Annex 2). In terms of cancer, only colorectal cancers and lung cancer were influenced by simulated dietary changes. The scenario is the same for both men and women in all the Nordic countries.

## 4. Discussion

In this model-based simulation study, we show that a considerable number of deaths could be prevented or delayed if the Nordic population adhere to the NNR. Among the Nordic Countries, Iceland would benefit the most by adhering to the NNR. We also find that the most lives could be saved by changes attributable to an increase in fruits and vegetable consumption except for Denmark, where most of the lives can be saved by reducing salt intake. Furthermore, it also revealed that more lives of men than women could be saved.

The simulation model predicts that the highest benefit would be gained for Iceland. This result is reasonable since Icelanders had the lowest intake of fiber and the highest intake of total fat, saturated fat, and a low intake of fruits and vegetables (Table 2). This result may be surprising since Iceland is one of the healthiest nations in the world according to the Bloomberg Healthiest Country index [33]. This index is based on several factors like health risks, availability of clean water, life expectancy, malnutrition, and causes of death where dietary habit is just one factor. Another reason might be that Icelanders have the lowest rate of physical inactivity among the Nordic countries [34], which is also a determinant of good health.

The highest number of deaths could be prevented or delayed by increasing the intake of fruits and vegetables for most countries where Finland would gain the most benefit. These findings are consistent with the Global Burden of Disease study, which revealed that health benefits are higher for food categories that are consumed in an insufficient amount, such as fruits and vegetables, than for foods and nutrients which are consumed in excess [35]. Findings for adolescent eating habits also indicated that fruit intake increased in Norway and Denmark, and the intake was the lowest for Finland [36]. In Denmark, a nation-wide 6-a-day initiative has been conducted since 2001 to increase the intake of fruits and vegetables in the population, which was effective in increasing the fruit and vegetable intake of the country [37]. In Norway, a free program of fruit in school (without parental payment) was implemented nationwide in 2007, which showed an increase in the consumption of fruits not only in school children but also in their parents [38]. It is worth mentioning that a maximum of one portion of fruit juice is considered as fruit in all the countries, except Iceland where any portion of fruit juice is included as fruit [19]. Therefore, the inclusion of fruit juice may have contributed to the mean intake of fruits and vegetables in the Nordic countries, which have an impact on our results. In order to achieve the Nordic Ambition, i.e., by 2021, at least 70% of the population complies with the NNR of a daily intake of 500 g of fruits and vegetables, there is an urgent need, for policy implications in Nordic countries, to increase the fruits and vegetables intake of the population [39].

The benefits from following the recommended salt intake are the third highest except for Denmark where it is the highest, according to the simulation model. For Denmark, the major portion of the salt comes from processed or semi-processed food, such as bread, meat, meat products, and cheese. Finland had the lowest salt intake among the Nordic countries. Since the 1970s, Finland has aimed to reduce salt intake in its National Nutrition Policy [40] by reformulation and raising public awareness of the harmful effects of salt on health. This has led to a significant reduction in salt intake of 3 g/day from 1979 to 2002 (12 to 9 g/day), as measured by urinary sodium [41], where the reduction was higher among women than men. The dietary survey (FINDIET’2012) [25] even provided a lower estimate of a 4.4 g/day reduction (from 12 to 7.6 g/day). This was accompanied by a fall in blood pressure and a decrease of 75%–80% in coronary heart disease and stroke mortality, with an increase of 5–6 years in life expectancy [42,43]. Finish action policy can be used as an example for other Nordic countries in terms of the reduction of salt intake. However, it is noteworthy that the sodium values are underestimated, as information on the addition of salt during cooking and at the dinner table was not systematically obtained during the diet interviews in Norway [26], as well as in the dietary surveys of Sweden [23] and Denmark [24]. Studies other than dietary surveys also indicate that salt consumption values might be underestimated in the dietary surveys [44,45,46]. This means that the effect we estimated by simulation is probably also underestimated. Another interesting fact is that the salt intake of elderly women from Finland (65–74 years) and Norway (60–70 years) was lower than the recommended level, 5.70 g/day and 5.75 g/day, respectively (Supplementary Materials). This means that even an intake lower than the recommended intake of 6 g/day is possible. The Nordic countries could consider lowering the recommended level, since the American Heart Association has suggested reducing salt intake to 3.75 g/day for the primary prevention of CVDs [47]. Furthermore, the European Food Safety Authority (EFSA) has recently positioned their assessment of sodium consumption for public consultation, where 3 g of sodium (equivalent to 5 g of salt) is suggested to reduce the risk of CVDs in the population [48].

The health benefits from changing the intake of fats and fatty acids are fewer compared to other dietary components. The estimates from the simulation model report on both the strength of the association between dietary factors and its health outcomes. The actual intake of fat and fatty acids for the Nordic population are very close to the recommended intake. Moreover, the population of the Nordic countries consumes a considerable number of dairy products and fish. Fish consumption has been measured to be the highest in Norway, followed by Iceland and Finland [20]. Fatty fishes are a good source of PUFA, and epidemiological studies suggested that diets rich in PUFA and MUFA are associated with low mortality [49,50].

It is noteworthy that women could gain the most from increased fiber intake, except in Iceland and Finland, whereas men could gain higher health benefits from an increased intake of fruits and vegetables. The reason for this result may be that men consume fewer servings of fruits and vegetables than women, whereas women consume less fiber than men (Table 2). Since a significant amount of fiber is are available from fruits and vegetables, this finding is questionable. One explanation is that fruit, vegetable, and fiber intake were separated in the simulation model. Moreover, the caloric intake of men was higher than women, and a significant portion of the calories came from grains, which are a high source of fiber, especially whole grains [51]. The most commonly consumed whole grain cereals in the Nordic countries are wheat, rye, and oats, with a considerable inter-country variation in consumption patterns [52], as well as inter-age group variation within the country. For example, rye bread is an important feature in the Danish diet [53]. In Sweden, older adults consume more whole grain products than younger adults [54].

The results from this study can be compared with studies that use the earlier version of the PRIME model to estimate the health impact of achieving dietary recommendations in Canada [30] and the UK [31]. The UK study suggested that 46% of the deaths averted or delayed could be attributed to meeting fruit and vegetable recommendations [31], with a further 23% attributed to achieving the salt recommendation. For Canada, it was 72% and 10% for fruits and vegetables and salt, respectively [30]. Sweden, Norway, and Iceland are close to the UK study for the fruit and vegetable intake, whereas Finland is close to the Canadian study for the salt intake. The reason for this result might be the difference between recommended fruits and vegetables consumption in the Nordic countries, Canada and the UK. The recommendation for Canada is at least seven servings (depending on sex and age) [30] and in the UK, it is five servings per day [55] (equivalent to 400 g), whereas we used the NNR which is 500 g per day [1]. The Nordic food culture is different from food cultures from the UK and Canada. Since the food culture, dietary practices, and recommendations are country-specific, a study on the health benefits for each Nordic country is justified. Meier et al. showed that 12 dietary factors contributed to 22.4% of all deaths in 51 European countries, based on the Global Burden of Disease study [56]. Our model-based simulation findings for Nordic countries are lower than that. One reason for this result could be that the PRIME model does not consider deaths related to processed meat and sugar and sweetened beverages.

The need for comparable data on nutrient intake across the Nordic countries is complicated due to diverse study methodologies. The methods used in the Nordic countries for the dietary surveys were different (Table 1). The 4 or 7 day consecutive surveys, 24 or 48 h recall, and food frequency questionnaire (FFQ) each have their pros and cons [57]. While 24 h recall suffers from underreporting, it is less onerous for the respondents [58]. Collection over more days better reflects usual intake due to greater control over day-to-day variation but is associated with within-person errors and cannot capture the wide variations of intake within the population. The FFQ can capture the inflated energy and nutrient intake but is burdensome for the respondent [59]. Given that the Nordic countries perform dietary surveys regularly, standardizing the survey methodology would vastly improve data comparability across the Nordic Countries. For example, both Norway and Iceland used 2 × 24 h recall together with an FFQ, which is recommended by the EFSA [60]. This can be a way forward for the harmonization of dietary surveys in the Nordic countries and could thus facilitate comparison.

Differences in dietary assessment methodologies present further limiting factors when making inter-country comparisons. For example, the mean energy intakes of Norwegian men aged 18–29 years were 3059 kcal per day [26], which is much higher than in the same age group in Sweden, (2246 kcal per day) [24], despite the fact that both national dietary were conducted in the same years (Supplementary Materials). These differences could thus result from either different methodological approaches to calculate the energy or a disparity in the intake.

The participation rates in the national dietary surveys vary to a large extent, and there is, in general, a low participation rate (Table 1). The highest participation rate was for Iceland (68.6%) [27]. Therefore, one cannot reject the notion of selection bias as only motivated people participated in the dietary surveys. Thus, the dietary surveys might not capture the true dietary intake of the population. Furthermore, underreporting is common and varies across methods and is affected by multiple other factors, making it difficult for comparison. For example, Norway excluded under-reporters, whereas Denmark included under-reporters in their analysis; other countries did not specify [61].

The lack of alignment and completeness of national nutrient databases and classification systems present further limitations. Nutrient databases are required to calculate energy and nutrient intakes from food consumption data and are prone to random and systematic dietary measurement errors, which can affect population means and the distribution of nutrient intakes [62]. An inter-country comparison is difficult due to a lack of harmonization of nutrients, i.e., modes of expression, units, and chemical analytical methods of analysis. For example, the Englyst method provides lower estimates of dietary fibers from certain cereals, fruits, white beans, and peanuts compared to the AOAC method [63]. A detailed description of different dietary surveys to estimate national dietary intake using different methods, nutrient databases, and the problem with inter-country comparison can be found elsewhere [64]. These differences are a major drawback for inter-country comparison, and thus our findings need to be interpreted with caution.

The strength of this study is that it uses the same simulation model, which facilitates cross-country comparison. The mortality statistics and population data were obtained from country-specific credible sources, and it is known that Nordic countries maintain good epidemiological data due to unique personal identification numbers and validated national registers [65]. These registers are well maintained with a high coverage rate [66], which is also a strength of the study. However, it is worth mentioning that, from the Danish statistical website, some of the mortality data required for the model input were unavailable (e.g., Heart failure, Aortic aneurysm), so the results might be underestimated for Denmark.

PRIME is a transparent model for which all the risk equations related to changes in diet and mortality come from high-quality meta-analyses [14]. Moreover, the model has been used several times in different countries (for example, in the UK [55,67,68], Ireland [69], New Zealand [70], France [29], and Canada [30]). The estimates of relative risks that have been used to parameterize the model were taken from the results of published meta-analyses, which is an additional strength of this study. However, not all of the studies included in the meta-analyses adjusted their results for each of the dietary factors or biological risk factors that are included in the model. For example, the effect of fruits and vegetables on CVDs is likely to be partially mediated by dietary fiber, which is not accounted for in the model [14]. The model-based findings may be affected by double counting to some extent. On these grounds, an overestimation of deaths prevented or delayed is possible, which is a limitation. Another limitation is that the analysis does not account for the health risks of consuming red meat. Furthermore, that the health benefits will be achieved in the same year if people follow NNR is an assumption of the model. It would, however, take years (e.g., the effects of salt reduction on CVDs) [71] or even decades (e.g., effects of dietary fiber on cancer) for the full health gain to be realized.

Nonetheless, we provide a comparative scenario in the Nordic countries in regard to discrepancies in terms of actual dietary intake and recommended dietary intake by NNR with a simulation model. This simulation model study has the potential for future research. We found that different countries require different areas for the policy implications of changes in dietary habits. Danes need to reduce their salt intake while Finns need to increase their fruit and vegetable intake. The next question would be to investigate how decision makers can intervene to modify the consumption of dietary components (e.g., increase fruit and vegetable intake or decrease salt intake by using taxes or subsidies [72,73,74] or by interventions at the workplace, for example, free fruit or healthy meals in the canteen [75,76,77]). A subsidy on grain products can modify the dietary fiber intake to the recommended level in the Swedish population [78,79]. The subsidy also resulted in an increased intake of other food components, such as fat, salt, and sugar. This indicates that both subsidies and taxes need to be used in order to modify the dietary behavior of the population [78,79]. For Denmark, a tax on saturated fat (16 Danish Krona per kilogram of saturated fat) reduced the intake of saturated fat by 4%, and at the same time, increased the consumption of vegetables, fruits, and fiber [80] in the population. Furthermore, Nordic countries can learn from each other on successful interventions/policies. For example, Denmark could benefit from the salt policy implemented in Finland, and the other Nordic counties may benefit from the 6-a-day campaign in Denmark, to increase fruit and vegetable intake.

## 5. Conclusions

In conclusion, this study shows that by modifying dietary intake, a considerable number of deaths could be prevented or delayed in the Nordic countries. Thus, policy makers should take the necessary steps to modify the dietary intake of the Nordic population.

## Figures and Tables

**Table 1:** National Dietary Survey across the Nordic Countries.

Country	Survey Name	Survey Year	Country Represent	Sample Age	Invited Sample	Sample Size	Participation Rate	Dietary Methodology	Nutrient Reference Database
Sweden [23]	Riksmaten 2010–2011 Swedish Adults Dietary Survey	2010–2011	Yes	18–80	5000	1797	36%	4 day food diary (consecutive)	The food database—Livsmedelsverket http://www7.slv.se/SokNaringsinnehall
Denmark [24]	Danish National Survey of Diet and Physical Activity (DANSDA) 2011–2013	2011 –2013	Yes	4–75	7253	3946	54.4%	7 day diary (consecutive)	Danish Food Composition Databank http://www.foodcomp.dk/v7/fcdb_default.asp
Finland [25]	The National FINDIET 2012 survey (FINRISK)	2012	No	25–74	3268	1708	52%	48 h recall	National Food Composition Database-Fineli https://fineli.fi/fineli/en/index
Norway [26]	Norwegian national diet survey NORKOST3	2010–2011	Yes	18–70	5000	1787	37%	2 × 24 h recall and FFQ	The Norwegian Food Composition Table http://www.matportalen.no/
Iceland [27]	The Diet of Icelanders—a national dietary survey 2010–2011	2010–2011	Yes	18–80	2000	1312	68.6%	2 × 24 h recall and FFQ	Icelandic Database of Food Ingredients (ÍSGEM); Public Health Institute for Raw Materials in the Icelandic Market http://www.matis.is/neytendur/leit-i-isgem-gagnagrunni/

Abbreviations: FFQ, Food Frequency Questionnaire; h, hour.

**Table 2:** Mean dietary component intake versus recommended intake (RI) for men and women in Nordic Countries.

Food/Nutrient	RI *	Sweden		Denmark		Norway		Finland		Iceland	
		Men (*n* = 792)	Women (*n* = 1005)	Men (*n* = 1494)	Women (*n* = 1552)	Men (*n* = 862)	Women (*n* = 925)	Men (*n* = 795)	Women (*n* = 913)	Men (*n* = 632)	Women (*n* = 6 80)
Fruits (g/day)	250	105.0 (3.97)	147.0 (3.53)	162.30 (3.74)	209.0 (3.58)	162.2 (5.06)	188.0 (4.63)	102.8 (5.05)	146.2 (5.54)	102.0 (4.73)	136.0 (4.69)
Vegetables (g/day)	250	169.0 (3.69)	182.0 (3.09)	190.33 (3.09)	204.5 (2.85)	156.2 (3.62)	153.4 (3.41)	83.0 (3.45)	92.6 (2.74)	121.0 (4.25)	110.0 (3.57)
Fiber (g/day)	25–35 (30)	21.30 (0.29)	18.80 (0.22)	28.83 (0.23)	20.83 (0.17)	26.6 (0.37)	22.2 (0.27)	22.0 (0.35)	20.6 (0.28)	17.8 (0.32)	15.83 (0.24)
Salt (g/day)	6	8.84 (0.10)	6.78 (0.063)	10.96 (0.08)	8.04 (0.06)	9.05 (0.12)	6.25 (0.08)	8.76 (0.11)	6.38 (0.07)	9.46 (0.15)	6.48 (0.09)
Total fat (%E)	25–40 (40)	34.0 (1.21)	34.40 (0.20)	36.33 (0.14)	35.83 (0.13)	34.0 (0.25)	34.2 (0.24)	35.84 (0.28)	35.14 (0.26)	36.53 (0.28)	35.43 (0.27)
Saturated fat (%E)	<10 (9)	13.0 (0.11)	13.10 (0.10)	14.5 (0.07)	13.83 (0.07)	13.0 (0.10)	13.4 (0.10)	13.8 (0.14)	13.52 (0.14)	14.57 (0.16)	14.13 (0.14)
MUFA (%E)	10–20 (20)	12.80 (0.09)	12.90 (0.09)	13.67 (0.06)	13.17 (0.06)	11.8 (0.10)	11.6 (0.10)	12.92 (0.13)	12.4 (0.12)	11.70 (0.09)	11.3 (0.10)
PUFA (%E)	5–10 (10)	5.5 (0.07)	5.7 (0.06)	5.52 (0.03)	5.65 (0.03)	6.22 (0.07)	6.16 (0.08)	6.20 (0.08)	6.26 (0.08)	5.87 (0.1)	5.9 (0.1)
Cholesterol (mg/day)	300	320 (5.15)	263 (3.9)	NA	NA	400.4 (0.76)	297 (5.58)	288.6 (6.16)	205.8 (3.84)	392 (8.10)	262 (4.83)

Abbreviations: RI, Recommended Intake; %E, percentage of total energy; MUFA, Monounsaturated fatty acids; NA, Not Available; PUFA, Polyunsaturated fatty acids. Note: Standard error of mean are in the parentheses. Recommended intakes are based on Nordic Nutrition Recommendation 2012. Recommended intake for fruits and vegetables together is 500 g/day, excluding fruit juice. The amount was divided equally for fruits and vegetables. * Recommended intakes for fiber and fatty acids are provided as range in the Nordic Nutrition Recommendations (NNRs), and a single value (in the parentheses) is used in the model simulation.

**Table 3:** Estimated number of total deaths or delayed by specific dietary changes according to guidelines in a year in the Nordic countries.

Country	Food Groups				All Dietary Guidelines Combined	Actual Death	% Averted by RI
	Fruits and Vegetables	Fiber	Fats	Salt			
Sweden							
Men	1905 (1262–2152)	718 (512–1275)	623 (471–792)	666 (335–1175)	3626 (2994–4175)	21,638	16.75%
Women	1073 (811–1420)	1285 (656–1577)	245 (224–487)	180 (63–237)	2553 (2030–2980)	22,816	11.18%
Altogether	3013 (2080–3566)	2025 (1197–2792	969 (709–1274)	1057 (391–1423)	6405 (5086–7086)	44,454	14.41%
Denmark							
Men	563 (406–725)	349 (196–502)	55 (11–99)	755 (326–1166)	1591 (1156–1997)	16,150	9.85%
Women	212 (136–288)	380 (219–545)	12 (7–33)	282 (122–447)	846 (623–1072)	16,418	5.15%
Altogether	773 (547–1002)	726 (413–1041)	67 (5–132)	1040 (453–1605)	2433 (1799–3053)	32,568	7.47%
Norway							
Men	584 (389–773)	324 (180–475)	126 (82–173)	391 (159–638)	1312 (1020–1605)	11,162	11.75%
Women	432 (265–591)	494 (296–688)	79 (46–120)	30 (5–76)	968 (739–1188)	12,271	7.89%
Altogether	1016 (662–1378)	820 (464–1163)	204 (132–289)	422 (171–727)	2285 (1786–2770)	23,433	9.75%
Finland							
Men	1985 (1357–2525)	845 (446–1248)	207 (119–297)	506 (212–800)	3141 (2517–3708)	14,549	21.59%
Women	1529 (1043–1975)	903 (512–1293)	37 (−22 – 99)	16 (4–38)	2286 (1776–2764)	14,097	16.21%
Altogether	3521 (2412–4503)	1747 (946–2541)	243 (101–396)	516 (207–850)	5421 (4280–6476)	28,646	18.92%
Iceland							
Men	68 (48–87)	51 (28–71)	20 (17–23)	28 (12–45)	141 (117–163)	586	24.06%
Women	46 (32–58)	37 (22–51)	7 (5–9)	2 (1–5)	81 (66–96)	543	14.9%
Altogether	114 (82–145)	88 (51–121)	27 (22–32)	31 (12–50)	223 (185–257)	1129	

Abbreviations: RI, Recommended Intake; note: 95% uncertainty intervals are provided in the parentheses. Due to the stochastic nature of the model, the total figure might not be the same for adding up male and female together. Actual death is the number of deaths for a year in the specific countries due to the diseases used in the simulation mode.

## Supplementary Materials

The following are available online at https://www.mdpi.com/2072-6643/11/6/1434/s1, Annex 1: Country-specific estimated number of deaths averted or delayed by adhering to Nordic Nutrition Recommendations and Annex 2: Country-specific age-group and sex-specific mean (standard deviation) dietary intake incorporated in the PRIME model
