# Supplementary material for: Modelling the Effect of Compliance with Nordic Nutrition Recommendations on Cardiovascular Disease and Cancer Mortality in the Nordic Countries
Source: Nutrients. 2019 Jun 25;11(6):1434. doi: 10.3390/nu11061434 (PMC6627195; doi:10.3390/nu11061434)
Supplement: Supplementary file 1 [file nutrients-11-01434-s001.pdf]

Annex 1. Country-specific age-group and sex-specific mean (standard deviation) dietary intake incorporated in the PRIME model

Table 1. Age-group and sex-specific mean (standard deviation) dietary intake of Swedish population incorporated in the PRIME model *.

**Dietary components**	**Men (n=792)**				**Women (n=1005)**			
**Age-group and sample size**	**18-30 (132)**	**31-44 (183)**	**45-64 (308)**	**65-80 (169)**	**18-30 (202)**	31-44 (247)	45-64 (358)	65-80 (198)
Energy (kcal/day)	2246 (830)	2343 (573)	2254 (674)	2083 (550)	1819 (538)	1820 (517)	1755 (510)	1703 (432)
Vegetables (gm/day)	142 (98)	174 (89)	176 (114)	169 (100)	162 (96)	189 (95)	192 (104)	178 (90)
Fruits (gm/day)	67 (88)	73 (87)	117 (124)	149 (110)	112 (95)	135 (104)	155 (111)	182 (107)
Fibre (gm/day)	18.6 (8.5)	21.3 (7.9)	21.8 (8.0)	22.5 (7.6)	17.3 (6.5)	18.5 (7.3)	19.3 (7.0)	20.0 (6.5)
Salt (gm/day)	9.12 (3.47)	9.55 (2.56)	9.09 (2.91)	8.03 (2.16)	6.92 (2.06)	7.19 (2.18)	6.89 (1.93)	6.36 (1.85)
Fat (%E)	33.9 (6.7)	34.6 (6.4)	33.8 (6.5)	33.6 (6.3)	34.6 (5.7)	34.7 (5.6)	34.7 (7.3)	33.2 (5.5)
Saturated fat (%E)	13.2 (3.4)	13.2 (3.2)	13.0 (3.2)	12.8 (3.2)	13.1 (2.8)	13.3 (3.1)	13.2 (3.7)	12.8 (2.9)
MUFA (%E)	12.8 (2.8)	13.1 (2.8)	12.7 (2.8)	12.5 (2.5)	13.1 (2.7)	13.0 (2.5)	12.9 (3.1)	12.3 (2.5)
PUFA (%E)	5.2 (1.9)	5.6 (2.0)	5.4 (1.8)	5.6 (2.2)	5.8 (2.1)	5.6 (1.9)	5.8 (2.0)	5.5 (1.7)
Cholesterol (mg/day)	314 (172)	319 (139)	322 (141)	321 (138)	245 (121)	257 (113)	274 (129)	269 (123)

* Obtained from Riksmaten 2010–2011 Swedish Adults Dietary Survey.

Table 2. Age-group and sex-specific mean (standard deviation) dietary intake of Danish population incorporated in the PRIME model*.

Dietary components	Men (n=1494)						Women (n=1552)					
Age-group and sample size	18-24 (170)	25-34 (190)	35-44 (253)	45-54 (297)	55-64 (292)	65-75 (292)	18-24 (176)	25-34 (185)	35-44 (289)	45-54 (318)	55-64 (322)	65-75 (262)
Energy (kcal/day)	2557 (815)	2772 (882)	2725 (686)	2725 (793)	2629 (719)	2581 (736)	1936 (600)	2103 (691)	2103 (528)	2032 (502)	1984 (502)	1984 (535)
Vegetables (gm/day)	171 (98)	210 (116)	204 (151)	191 (101)	186 (109)	180 (142)	179 (94)	211 (105)	217 (123)	216 (119)	210 (116)	194 (116)
Fruits (gm/day)	131 (126)	147 (135)	139 (127)	176 (163)	184 (154)	197 (162)	188 (135)	183 (135)	197 (143)	214 (138)	220 (133)	250 (162)
Fibre (gm/day)	22 (9.4)	23 (8.5)	24 (8.8)	25 (8.2)	25 (8.4)	24 (9.1)	19 (7.2)	21 (7)	21 (6.6)	21 (6.6)	22 (6.3)	21 (7)

Salt (gm/day)	11 (3.7)	11.5 (3.35)	11.25 (2.98)	11.25 (3.28)	10.75 (3.18)	10 (3)	7.75 (2.5)	8.5 (2.7)	8.5 (2.2)	8 (2.3)	8 (2.2)	7.5 (2.2)
Fat (%E)	36 (6.1)	36 (4.9)	36 (4.9)	37 (5.5)	37 (5.6)	36 (5.5)	35 (5.3)	36 (4.6)	36 (4.9)	36 (5.4)	36 (5.6)	36 (5.5)
Saturated fat (%E)	14 (3)	14 (2.6)	14 (2.5)	15 (2.7)	15 (3.1)	15 (3.1)	13 (2.6)	14 (2.4)	14 (2.5)	14 (2.8)	14 (2.9)	14 (3.1)
MUFA (%E)	14 (2.9)	14 (2.2)	14 (2.3)	14 (2.5)	13 (2.4)	13 (2.3)	13 (2.5)	14 (2.1)	13 (2.3)	13 (2.6)	13 (2.5)	13 (2.3)
PUFA (%E)	5.6 (1.3)	5.5 (0.9)	5.5 (0.9)	5.5 (1)	5.5 (1)	5.5 (1)	5.5 (1.1)	5.7 (1.)	5.7 (1)	5.7 (1.2)	5.7 (1.2)	5.6 (1.0)
Cholesterol (mg/day)	NA	NA	NA	NA	NA	NA	NA	NA	NA	NA	NA	NA

NA, Not Available * Obtained from Danish National Survey of Diet and Physical Activity (DANSDA) 2011–2013

Table 3. Age-group and sex-specific mean (standard deviation) dietary intake of Norwegian population incorporated in the PRIME model* .

Dietary components	Men (n=862)					Women (n=925)				
Age-group and sample size	18-29 (138)	30-39 (136)	40-49 (179)	50-59 (192)	60-70 (217)	18-29 (143)	30-39 (169)	40-49 (256)	50-59 (193)	60-70 (164)
Energy (kcal/day)	3059 (956)	2749 (836)	2533 (741)	2486 (741)	2366 (693)	1936 (597)	2001 (574)	1936 (574)	1888 (550)	1769 (526)
Vegetables (gm/day)	178 (123)	153 (100)	151 (105)	158 (109)	141(94)	138 (87)	154 (121)	164 (105)	154 (91)	157 (114)
Fruits (gm/day)	131 (137)	133 (139)	153 (144)	179 (146)	215 (177)	146 (124)	178 (148)	178 (128)	205 (153)	233 (151)
Fibre (gm/day)	29 (12)	26 (12)	25 (10)	26 (10)	27 (11)	21 (8)	24 (10)	22 (8)	22 (7)	22 (8)
Salt (gm/day)	10 (4)	10 (4)	8.7 (3.25)	8.7 (3.25)	7.75 (3.25)	6.25 (2.5)	6.5 (2.7)	6.5 (2.2)	6.25 (2.5)	5.75 (2.5)
Fat (%E)	33 (6)	34 (8)	35 (7)	34 (8)	34 (8)	33 (7)	33 (7)	35 (7)	35 (8)	35 (7)
Saturated fat (%E)	13 (3)	13 (3)	13 (3)	13 (3)	13 (3)	13 (3)	13 (3)	14 (3)	13 (3)	14 (4)
MUFA (%E)	11 (3)	12 (3)	12 (3)	12 (3)	12 (3)	11 (3)	11 (3)	12 (3)	12 (3)	12 (3)
PUFA (%E)	5.9 (2.1)	6.1 (2.1)	6.6 (2.3)	6.3 (2.1)	6.2 (2.4)	5.8 (2.5)	5.9 (2.1)	6.3 (2.4)	6.4 (2.3)	6.4 (2.3)
Cholesterol (mg/day)	441 (283)	404 (229)	362 (179)	413 (229)	382 (199)	254 (183)	270 (138)	315 (183)	328 (166)	318 (179)

*Obtained from Norwegian national diet survey NORKOST3.

Table 4. Age-group and sex-specific mean (standard deviation) dietary intake of Finnish population incorporated in the PRIME model* .

Dietary components	Men (n=795)					Women (n=913)				
Age-group and sample size	25-34 (106)	35-44 (143)	45-54 (162)	55-64 (174)	65-74 (210)	25-34 (149)	35-44 (192)	45-54 (178)	55-64 (191)	65-74 (203)
Energy (kcal/day)	2449 (830)	2275 (573)	2282 (674)	2053 (550)	1954 (632)	1861 (491)	1832 (556)	1677 (456)	1586 (430)	1484 (423)
Vegetables (gm/day)	82 (88)	79 (79)	84 (98)	88 (118)	82 (103)	97 (83)	89 (75)	95 (92)	195 (91)	87 (73)
Fruits (gm/day)	51 (92)	82 (139)	95 (141)	149 (180)	137 (160)	132 (181)	117 (132)	150 (163)	169 (197)	163 (164)
Fibre (gm/day)	19 (8)	21 (11)	23 (10)	23 (10)	24 (11)	19 (9)	20 (9)	21 (7)	22 (9)	21 (9)
Salt (gm/day)	9.5 (3)	8.8 (2.9)	9.3 (3.7)	8.3 (3)	7.9 (2.7)	6.7 (2.3)	6.8 (2.2)	6.4 (2.1)	6.3 2.7)	5.7 (1.7)
Fat (%E)	36.7 (8)	35.6 (8.7)	35.9 (7.4)	36.4 (8.7)	34.6 (6.9)	36.5 (7.6)	36.1 (8)	34.8 (8.2)	34.8 (8.2)	33.5 (7.6)
Saturated fat (%E)	14.4 (4.1)	13.7 (4.3)	13.9 (3.9)	14 (4.5)	13 (3.5)	14.3 (4.3)	14 (4.1)	13.6 (4.4)	13 (3.9)	12.7 (4.1)
MUFA (%E)	13.3 (3.3)	13 (3.8)	13 (3.6)	12.9 (3.9)	12.4 (3.3)	12.8 (3)	13.0 (3.7)	12.3 (3.5)	12.3 (3.9)	11.6 (3.4)
PUFA (%E)	6.2 (2.4)	6 (2.2)	6.2 (2.3)	6.4 (2.5)	6.2 (2.2)	6.3 (2.4)	6.3 (2.4)	6.1 (2.1)	6.4 (2.6)	6.2 (2.8)
Cholesterol (mg/day)	336 (190)	293 (162)	302 (210)	270 (167)	242 (140)	227 (127)	223 (120)	210 (127)	185 (104)	184 (102)

* Obtained from The National FINDIET 2012 survey (FINRISK).

Table 5. Age-group and sex-specific mean (standard deviation) dietary intake of Icelandic population incorporated in the PRIME model* .

Dietary components	Men (n=632)			Women (n=680)		
Age-group and sample size	18-30 (131)	31-60 (350)	61-80 (151)	18-30 (119)	31-60 (394)	61-80 (167)
Energy (kcal/day)	2635 (883)	2402 (736)	2081 (678)	1895 (500)	1795 (536)	1610 (482)
Vegetables (gm/day)	118 (94)	125 (90)	120 (137)	106 (103)	130 (101)	94 (75)
Fruits (gm/day)	93 (121)	98 (117)	115 (119)	132 (137)	136 (118)	140 (112)
Fibre (gm/day)	19.1 (9.5)	17.6 (7.3)	16.7 (7.5)	16.2 (6.7)	16.5 (6.1)	14.8 (5.7)
Salt (gm/day)	10.14 (4.14)	9.44 (3.48)	8.8 (3.88)	6.69 (2.05)	6.57 (2.14)	6.18 (2.62)
Fat (%E)	33.6 (7)	36.6 (7.5)	39.4 (6.7)	32.8 (6.8)	36.1 (6.7)	37.4 (7.6)
Saturated fat (%E)	13 (3.4)	14.5 (4.2)	16.2 (4.2)	12.7 (3.6)	14.4 (3.4)	15.3 (3.9)
MUFA (%E)	10.9 (2.4)	11.8 (2.5)	12.6 (2.3)	10.7 (2.6)	11.4 (2.5)	11.8 (2.8)

PUFA (%E)	5.7 (2.3)	6 (2.5)	5.9 (2.3)	5.8 (2.7)	6.1 (2.5)	5.8 (2.3)
Cholesterol (mg/day)	388 (230)	390 (182)	398 (198)	224 (116)	275 (138)	287 (124)

* Obtained from The Diet of Icelanders—a national dietary survey 2010–2011.

Annex 2 Country-specific estimated number of deaths averted or delayed by adhering to Nordic Nutrition Recommendations

Table 1. Estimated numbers of deaths averted or delayed by cause if Swedish men and women adhered to Nordic Nutrition Recommendations.

Causes of death	Men (mean, 95% CI)	Women (mean, 95% CI)	Total (mean, 95% CI)
Cardiovascular diseases			
Coronary heart disease	2532 (1913 to 2775)	1623 (1266 to 2028)	4077 (4529 to 6462)
Stroke	551 (407 to 777)	609 (302 to 822)	1219 (729 to 1548)
Heart failure	72 (37 to 139)	30 (9 to 36)	147 (46 to 175)
Aortic aneurysm	27 (12 to 47)	4 (2 to 6)	49 (14 to 54)
Pulmonary embolism	6 (2 to 11)	1 (0 to 3)	10 (2 to 14)
Rheumatic Heart disease	1 (0 to 3)	0 (0 to 1)	2 (1 to 4)
Hypertensive disease	120 (59 to 200)	54 (18 to 65)	233 (77 to 267)
Cancer			
Colo-rectal	76 (10 to 241)	90 (32 to 178)	261 (96 to 363)
Lung	240 (103 to 381)	142 (45 to 174)	407 (141 to 564)

Table 2. Estimated numbers of deaths averted or delayed by cause if Danish men and women adhered to Nordic Nutrition Recommendations.

Causes of death	Men (mean, 95% CI)	Women (mean, 95% CI)	Total (mean, 95% CI)
Cardiovascular diseases			
Coronary heart disease	715 (535 to 879)	367 (245 to 483)	1075 (773 to 1346)
Stroke	349 (240 to 453)	260 (140 to 377)	610 (395 to 816)
Heart failure	196 (82 to 313)	77 (33 to 126)	274 (119 to 438)
Aortic aneurysm	14 (6 to 23)	5 (2 to 9)	19 (8 to 32)
Pulmonary embolism	31 (10 to 63)	9 (3 to 18)	40 (13 to 79)
Rheumatic Heart disease	2 (1 to 4)	1 (0 to 2)	3 (1 to 6)
Hypertensive disease	135 (59 to 204)	68 (29 to 108)	204 (89 to 311)
Cancer			

Colo-rectal	25 (2 to 49)	15 (4 to 26)	40 (15 to 66)
Lung	123 (47 to 199)	42 (15 to 72)	164 (65 to 274)

Table 3. Estimated numbers of deaths averted or delayed by cause if Finnish men and women adhered to Nordic Nutrition Recommendations.

Causes of death	Men (mean, 95% CI)	Women (mean, 95% CI)	Total (mean, 95% CI)
	Cardiovascular diseases		
Coronary heart disease	2334 (1778 to 2843)	1618 (1127 to 2071)	3968 (2868 to 4903)
Stroke	465 (338 to 587)	518 (316 to 700)	978 (654 to 1286)
Heart failure	2 (1 to 3)	0 (-1 to 0)	1 (0 to 2)
Aortic aneurysm	17 (7 to 28)	-1 (-1 to 0)	16 (7 to 27)
Pulmonary embolism	1 (0 to 2)	0 (-1 to 0)	1 (0 to 1)
Rheumatic Heart disease	0 (0 to 1)	0	0
Hypertensive disease	103 (43 to 165)	-19 (-10 to -22)	84 (34 to 144)
	Cancer		
Colo-rectal	56 (4 to 112)	45 (12 to 78)	102 (41 to 165)
Lung	160 (63 to 257)	71 (29 to 112)	230 (93 to 369)

Table 4. Estimated numbers of deaths averted or delayed by cause if Norwegian men and women adhered to Nordic Nutrition Recommendations.

Causes of death	Men (mean, 95% CI)	Women (mean, 95% CI)	Total (mean, 95% CI)
Cardiovascular diseases			
Coronary heart disease	800 (603 to 982)	642 (446 to 824)	1446 (1063 to 1807)
Stroke	193 (126 to 258)	200 (82 to 317)	393 (221 to 565)
Heart failure	74 (30 to 124)	-8 (-5 to -9)	66 (26 to 116)
Aortic aneurysm	0	0	0
Pulmonary embolism	0	0	0
Rheumatic Heart disease	0	0	0
Hypertensive disease	136 (54 to 228)	-2 (-5 to 16)	135 (50 to 244)
Cancer			
Colo-rectal	39 (2 to 84)	52 (15 to 87)	92 (38 to 149)
Lung	70 (27 to 114)	33 (12 to 55)	102 (40 to 168)

Table 5. Estimated numbers of deaths averted or delayed by cause if Icelandic men and women adhered to Nordic Nutrition Recommendations.

Causes of death	Men (mean, 95% CI)	Women (mean, 95% CI)	Total (mean, 95% CI)

Cardiovascular diseases			
Coronary heart disease	96 (77 to 113)	51 (38 to 62)	147 (116 to 174)
Stroke	20 (14 to 25)	17 (10 to 24)	37 (24 to 49)
Heart failure	4 (2 to 7)	0	5 (2 to 8)
Aortic aneurysm	1 (0 to 1)	0	1 (0 to 1)
Pulmonary embolism	0 (0 to 1)	0	0 (0 to 1)
Rheumatic Heart disease	0	0	0
Hypertensive disease	4 (2 to 6)	1 (0 to 2)	5 (2 to 8)
Cancer			
Colo-rectal	6 (0 to 11)	4 (1 to 7)	10 (4 to 16)
Lung	9 (4 to 14)	8 (3 to 13)	18 (6 to 28)
